# The influence of maternal health factors including multimorbidity on child oral health: A scoping review and evidence gap map protocol

**DOI:** 10.12688/wellcomeopenres.21725.1

**Published:** 2024-06-10

**Authors:** Faith Campbell, Scott McGregor, Louise Marryat, Ryan Stewart, Jan Clarkson, Heather Cassie

**Affiliations:** 1 School of Dentistry, University of Dundee, Dundee, Scotland, UK; 2 Mother, Infant and Child Research Group, School of Health Sciences,, University of Dundee, Dundee, Scotland, UK; 3 Department of Mathematics and Statistics, University of Strathclyde, Glasgow, Scotland, UK

**Keywords:** Oral health, dental caries, maternal health

## Abstract

**Objective:**

The objective of this scoping review is to map the extent and type of evidence in relation to the association between maternal health conditions, including multimorbidity and child oral health.

**Introduction:**

Child oral health research has historically focussed on toothbrushing, diet and neglect of care, including not taking children to dental appointments. Newer theories are emerging that detail the many factors that can influence child oral health at child, family and community levels ^1^. More recently, the association between maternal general health and child oral health has been explored, with preliminary evidence suggesting a link between shared environmental factors and direct maternal-to-child transfer of bacteria and diet/substance use during pregnancy causing childhood caries ^2, 3^.

**Inclusion criteria:**

This review will include all published studies that describe the relationship between maternal health (including multimorbidity) and child oral health. There will be no limitation on the date of publication due to the limited number of studies available from the initial search of PubMed. The review will exclude case studies, abstracts, and grey literature. Literature must be in English language.

**Methods:**

The following databases will be searched; CINAHL, Cochrane Library, Maternity and Infant Care, Medline via PubMed, Scopus, Web of Science. The search will include sources in English only and will be undertaken between April and July 2024. Studies to be included will be of any type of study design that describe a relationship between maternal health factors, including maternal oral health, and child oral health. Data extraction will be undertaken using tabulation of results by at least two independent reviewers. Narrative analysis of the evidence will be undertaken, and results will be presented in a narrative and tabular manner due to the heterogenous and limited evidence base found in the test search. This review has been registered prospectively on Open Science Framework, ( https://doi.org/10.17605/OSF.IO/ECSWJ). The review will also inform an Evidence Gap Map (EGM) to illustrate the current evidence base regarding maternal health factors that influence child oral health.

## Introduction

This review will be part of Faith Campbell’s PhD entitled ‘Intergenerational multimorbidity and pathways to oral health in early childhood’. To explore this relationship, it is important to map the current evidence base on the concept of maternal health and child oral health.

### Multimorbidity

Multimorbidity is the presence of two or more co-existing chronic health conditions, whereas a co-morbidity is an additional chronic health condition in relation to a primary health condition which infers greater importance to the primary health condition. Multimorbidity affects approximately 37% of adults globally and over the last two decades the prevalence has increased ^4^. Multimorbidity is a key challenge facing healthcare systems ^5^.

Multimorbidity significantly affects women of childbearing age, with a recent epidemiological study in Scotland finding that one in six mothers lived with multimorbidity prior to their pregnancy ^6^. The term ‘maternal’ will be used throughout this work to describe the population of interest more precisely as data will only be available for mothers in the work that this review will inform. The authors are aware that the term ‘childbearing people’ is more inclusive, however feel that this introduces ambiguity to search terms therefore it will be avoided.

### Dental caries

Dental caries (dental decay) is preventable and is the most prevalent disease worldwide, affecting 2.4 billion people ^7^. It has a significant impact and when severe can impact quality of life, for example causing abscesses, difficulties eating and sleeping and may result in pain, chronic infection or failure to thrive ^8^. Dental caries has a linear relationship with poverty, affecting lower socio-economic groups the most ^9^. In Scotland in 2020, 58.1% of children aged 5 living in the 20% most deprived areas showed no obvious caries experience, compared with 86.9% of children aged 5 in the 20% least deprived areas ^9^. Dental extractions are the most common reason for elective admittance to hospital for general anesthesia for children in Scotland and England, costing the National Health Service in Scotland approximately £5million per year ^10, 11^.

### Possible link

There has been a demonstrated link between poor oral health, socio-economic inequalities and co-morbidities including diabetes, cardiovascular disease, rheumatoid arthritis, chronic obstructive pulmonary disease (COPD), pneumonia in the elderly, anxiety and depressive disorders, and increased risk of developing cognitive impairment and dementia ^12^. This link is frequently bi-directional. However, the link between multimorbidity and oral health, particularly dental caries, is less researched ^12^. Multimorbidity and poor oral health share some common risk factors such as deprivation: both dental caries and multimorbidity disproportionately affect those living in more deprived areas ^9, 13^.

The increasing number of people living with multimorbidity, results in an increasing need for new models of dental care to address multimorbidity in relation to adult oral health ^12^. Individuals with multimorbidity have a greater treatment burden than the general population, whereby they need to access treatment from multiple (often uncoordinated) healthcare professionals ^12^. These care plans are often complex and those with multimorbidity are often taking multiple medications ^12^. Best care for these patients requires appropriate training of oral health professionals, changes to the practice and delivery of oral health care and a focus of research on multimorbidity and oral health ^12^.

### Current evidence

Initial explorations indicate that there is a limited but evolving body of evidence regarding child oral health and child or maternal multimorbidity. Newer theoretical models are emerging that detail the many factors that can influence child oral health at child, family and community levels ^1^. The association between maternal multimorbidity and child oral health has been explored, with preliminary evidence suggesting a link between shared environmental factors and direct maternal-to-child transfer of bacteria, diet and substance use during pregnancy causing childhood caries ^2, 3^. Maternal multimorbidity may additionally create challenges in supporting child oral care and in attending dental appointments ^1^. To date, no review has synthesised this evidence to examine the relationship between maternal multimorbidity and child oral health.

### Importance of research

Oral health is a priority at a global and national level within Scotland ^10, 14, 15^. Given the importance of good oral health and the demonstrable impacts that poor oral health has on children, their families and society, mapping the current concepts regarding the relationship between maternal multimorbidity and child oral health will help to inform future research and targeted health and social care resource delivery.

Scoping reviews usually have a broader scope and less restrictive inclusion criteria than systematic reviews and are useful in mapping out current concepts and evidence such as in emerging fields where evidence is more limited ^16^. A scoping review has been chosen in this case because it is the most appropriate method to achieve the purpose of this review which is to map the key concepts within this emerging evidence base ^17^. This will allow for the identification and discussion of current concepts relating to maternal multimorbidity and child oral health. The review will follow systematic methods.

A preliminary search of MEDLINE, the Cochrane Database of Systematic Reviews, *JBI Evidence Synthesis and* Open Science Framework was conducted and no current or underway systematic reviews or scoping reviews on the topic were identified.

The objective of this scoping review is to map existing literature on the relationship between maternal health factors, including multimorbidity, and child oral health. Maternal health factors have been chosen because an initial pilot search found such limited evidence regarding maternal multimorbidity as a concept, that a broader search to map the evidence base on maternal health factors and child oral health would provide greater value.

## Review question

### Primary question

What is the current evidence base regarding the relationship between maternal health factors and child oral health?

### Additional question(s)

Is maternal oral health included as a condition when assessing maternal health in relation to child oral health?

Does the current evidence base describe a link between maternal multimorbidity and maternal oral health?

Does the current evidence base describe a link between maternal oral health and child oral health?

What are the current theories linking maternal health factors including multimorbidity and child oral health globally?

## Eligibility criteria

The CoCoPop mnemonic has been used, which is recommended for reviews that explore epidemiological data such as prevalence and incidence, where the traditional PICO mnemonic is not as applicable ^18^. CoCoPop describes the condition, context, and population ^18^.

### Population

Inclusion criteria: maternal health data at any time from prior to conception to the child being 16 years old.

Exclusion: Evidence not related to health of mothers.

### Condition

Inclusion criteria: single or multiple health conditions (multimorbidity) affecting mothers at any time from prior to conception to the child being 16 years old.

Exclusion: No clear definition of health condition that is affecting mother.

### Context

All settings and countries will be included. Only studies published in English will be included.

Exclusion: studies not published in English.

### Types of sources

This scoping review will consider both experimental and quasi-experimental study designs including randomized controlled trials, non-randomized controlled trials, before and after studies and interrupted time-series studies. In addition, analytical observational studies including prospective and retrospective cohort studies, case-control studies and analytical cross-sectional studies will be considered for inclusion. This review will also consider descriptive observational study designs including case series and descriptive cross-sectional studies for inclusion.

Furthermore, systematic reviews that meet the inclusion criteria will also be considered and the studies within those reviews cross-referenced with the results from the database searches to avoid duplication of evidence.

This review will not include individual case studies and abstracts.

Eligibility criteria will be reviewed and refined if appropriate following undertaking the first search.

## Methods

The proposed protocol has been registered with Open Science Framework ( https://doi.org/10.17605/OSF.IO/ECSWJ). The proposed scoping review will be conducted in accordance with the JBI methodology for scoping reviews ^17^.

### Search strategy

The search strategy will aim to locate the published literature on this topic. An initial limited search of Medline via PubMed was undertaken to identify articles on the topic. The text words contained in the titles and abstracts of relevant articles, and the index terms used to describe the articles were used to develop a full search strategy for *CINAHL, Cochrane Library, Maternity and Infant Care, Medline via PubMed, Scopus and Web of Science, Table 1*. The search strategy, including all identified keywords and index terms, will be adapted for each included database and/or information source. The reference list of all included sources of evidence will be screened for additional studies.

**Table 1.** Search strategy for Medline (via PubMed).

Search	Query
1.	Child[Mesh]
2.	Child, Preschool[Mesh]
3.	Infant[Mesh]
4.	Infant[Title/Abstract]
5.	Child*[Title/Abstract]
6.	or/1-5
7.	Oral Health[Mesh]
8.	Oral Health[Title/Abstract]
9.	Dental Health[Title/Abstract]
10.	Dental Caries[Mesh]
11.	"Dental Caries"[Title/Abstract]
12.	"Early childhood caries"[Title/Abstract]
13.	DMF Index[Mesh]
14.	or/7-13
15.	Mothers[Mesh]
16.	Maternal health[Title/Abstract]
17.	Parental health[Title/Abstract]
18.	Maternal factors[Title/Abstract]
19.	Maternal Behavior[Mesh]
20.	Mother* health[Title/Abstract]
21.	Pregnancy[Mesh]
22.	Prenatal[Title/Abstract]
23.	Antenatal[Title/Abstract]
24.	Neonatal[Title/Abstract]
25.	Postnatal[Title/Abstract]
26.	or/15-25
27.	6 and 14 and 26

Studies published in English will be included. There will be no limitation on the date of publication due to the limited number of studies available from the initial search of PubMed.

The databases to be searched include ( *CINAHL, Cochrane Library, Maternity and Infant Care, Medline via PubMed, Scopus, Web of Science)*. Where appropriate authors will be contacted for further information. Sources of unpublished studies/grey literature will not be searched.

### Study/source of evidence selection

Following the search, all identified citations will be uploaded into Covidence systematic review software (Covidence systematic review software, Veritas Health Innovation, Melbourne, Australia. Available at www.covidence.org) and duplicates removed. Following a pilot screening, titles and abstracts will then be screened separately by two or more independent reviewers for assessment against the inclusion criteria for the review. Potentially relevant sources will be retrieved in full, and their citation details imported into Covidence systematic review software, Veritas Health Innovation, Melbourne, Australia (Available at www.covidence.org). The full text of selected citations will be assessed in detail against the inclusion criteria by two or more independent reviewers. Reasons for exclusion of sources of evidence at full text that do not meet the inclusion criteria will be recorded and reported in the scoping review. Any disagreements that arise between the reviewers at each stage of the selection process will be resolved through discussion, or with an additional reviewer/s. The results of the search and the study inclusion process will be reported in full in the final scoping review and presented in a Preferred Reporting Items for Systematic Reviews and Meta-analyses extension for scoping review (PRISMA-ScR) flow diagram ^19^ *.*

### Data extraction

Data will be charted from papers included in the scoping review by two or more independent reviewers using a data extraction tool developed by the reviewers, Table 2. This form has been piloted against two known papers that discuss maternal health (or multimorbidity) and child oral health by two independent reviewers who then met to discuss and refine the form. The data extracted will include specific details about the participants, concept, context, study methods and key findings relevant to the review question/s.

**Table 2.** Data extraction tool.

Scoping Review Details	
Scoping Review title:	
Review objective/s:	
Review question/s:	
Inclusion/Exclusion Criteria	
Population	
Concept	
Context	
Types of evidence source	
Evidence source Details and Characteristics	
Citation details (e.g. author/s, date, title, journal, volume, issue, pages)	
Country	
Context	
Participants (details e.g. age/sex and number)	
Type of study	
**Details/Results extracted from source of evidence** (in relation to the concept of the scoping review)	
If multimorbidity is included:	
Reference definition of multimorbidity	
Prevalence of maternal multimorbidity described	
Type of multimorbidity measure (weighted or count)	
Data source for multimorbidity (self report, medical records, administrative database)	
Number of conditions included in the multimorbidity measure, and the actual conditions included	
Which conditions are included when describing maternal multimorbidity?	
Is maternal oral health included as a condition when assessing maternal multimorbidity in relation to child oral health?	
Does source describe a link between maternal multimorbidity and maternal oral health?	
Theory linking maternal multimorbidity and child oral health	
Other maternal health conditions:	
Maternal health condition discussed and definition of this from source (if not multimorbidity)	
Does this health condition influence child oral health?	
Theory on mechanism of action for this health condition influencing child oral health	
Maternal oral health:	
Does source describe a link between and maternal oral health and child oral health?	
Theory linking maternal oral health and child oral health	
Other influencing factor(s):	
Presence of PROGRESS – Plus? Yes/No	
Which PROGRESS -plus factors?	
Other maternal influencing factor on child oral health	
Theory on mechanism of action for this influencing factor	
Coding for EGM	
Exposure (please see definitions in Table 3)	
Outcome (please see definitions in Table 3)	
Quality of evidence (risk of bias)	

### Quality appraisal

Although quality appraisal is not generally undertaken for scoping reviews, it will be in this case due to the evidence identified being included in an EGM. It is recommended that quality appraisal is undertaken for evidence included in EGMs ^21^. Quality appraisal will be undertaken using the appropriate tool for each type of study, for example the Cochrane risk of bias (RoB 2) tool ^22^.

### Data analysis and presentation

Once all sources have been identified, data characteristics will be summarised, data will be reported and interpreted by the research team. Simple frequency counts and descriptive analysis will be used for this review. This is because the evidence base of interest is heterogenous and limited. Data will be presented in tabular form and if suitable graphically. A narrative summary will accompany the tabulated and/or charted results and will describe how the results relate to the reviews objective and questions.

### Evidence Gap Mapping

Evidence Gap Maps (EGM) are a systematic evidence synthesis product which display the available evidence relevant to a specific research question ^21^ (4). An EGM can demonstrate areas for further research both with respect to the quality and quantity of evidence available. This is a novel method for illustrating gaps in the evidence base which has rarely been used in oral health.

EGMs are based on a framework of interventions and outcomes, which is developed prior to the review with stakeholder involvement ^21^. For this scoping review which will explore exposures and outcomes, the Campbell framework will be adapted to include exposures, rather than the traditional interventions and outcome ^21^. Frameworks should be based on policy documents ^21^. Oral health is a key priority for the World Health Organisation (WHO), and this is described within global strategy on oral health adopted at World Health Assembly 75 ^14^. The WHO describes five key oral diseases as ^23^;

- Caries of deciduous teeth
- Caries of permanent teeth
- Severe periodontal disease
- Edentulism
- Lip and oral cavity cancer

Additionally, the following conditions are described as part of oral health ^23^;

- Oral manifestation of systemic disease
- Oral mucosa disease
- Erosion and tooth wear
- Oral impacts of substance misuse
- Noma
- Congenital malformations of teeth and enamel
- Viral, bacterial and fungal infections
- Trauma (including physical and chemical injuries) of the teeth, jawbones and adjacent maxillofacial structures
- Cysts and tumours of odontogenic origin
- Salivary gland disease
- Disturbances in the development and growth of oral structures

This list provides a framework for oral health outcomes, which will be adapted to exclude conditions that do not affect children and will be reviewed by subject experts and PPI advisors.

As this study does not include an intervention, then exposures, being maternal conditions, will be considered instead. This will include the following factors;

- Pre-natal maternal health conditions
  ⚬ Single condition
  ⚬ Multiple conditions
    ▪ Single condition dominant (co-morbidity)
    ▪ Two or more conditions of equal importance (multimorbidity)
- Perinatal complications
- Post natal maternal health conditions
  ⚬ Single condition
  ⚬ Multiple conditions
    ▪ Single condition dominant (co-morbidity)
    ▪ Two or more conditions of equal importance (multimorbidity)
- No specified time for maternal conditions
- Lifestyle factors
  ⚬ Tobacco
  ⚬ Alcohol
- Genetic factors
- Bacterial factors
- Other including PROGRESS-Plus factor

As this is a novel study and application of an EGM the framework will be adapted once the scoping review is complete to include any exposures that may arise and are not described above. This will be undertaken in consultation with subject experts and PPI advisors. The initial planned framework is included in Table 3.

**Table 3.** Data charting tool for the evidence gap map.

	Outcome	Caries of primary teeth	Caries of permanent teeth	Severe periodontal disease	Edentulism	Oral manifestation of systemic disease	Oral mucosa disease	Erosion and tooth wear	Noma	Congenital malformations of teeth and enamel	Viral, bacterial and fungal infections	Trauma (including physical and chemical injuries) of the teeth, jawbones and adjacent maxillofacial structures	Cysts and tumours of odontogenic origin	Salivary gland disease	Disturbances in the development and growth of oral structures
Exposure															
Pre-natal maternal health conditions															
Single condition															
Comorbidity															
Multimorbidity															
Perinatal complications															
Post-natal maternal health conditions															
Single condition															
Co-morbidity															
Multimorbidity															
Maternal health conditions with no specified time															
Single condition															
Co-morbidity															
Multimorbidity															
Maternal lifestyle factors															
Tobacco consumption															
Alcohol consumption															
Genetic factors															
Bacterial factors															
PROGRESS – plus factor															
Other															

The scoping review will locate the available evidence on the question. This will then be coded using the appropriate column in the framework in Table 4, with respect to any relevant exposure on maternal health factors and outcome on child oral health outcomes. Should the evidence not fit into an existing code then the existing codes will be expanded to include the evidence, as recommended in the EGM guidance ^21^.

**Table 4.** Coding terms definitions.

Coding term	Definition
Caries of deciduous teeth	Dental decay affecting the first set of teeth
Caries of permanent teeth	Dental decay affecting adult teeth, from time of formation/eruption which is in childhood
Severe periodontal disease	Gum disease which has resulted in significant bone loss or tooth loss
Edentulism	The absence of teeth
Lip and oral cavity cancer	Malignancy affecting and structures in the oral cavity
Oral manifestation of systemic disease	Oral conditions that can be attributed to a systemic disease
Oral mucosa disease	Any condition affecting the mucous membrane within the oral cavity
Erosion and tooth wear	Non carious tooth surface loss affecting the primary and/or permanent teeth
Noma	Necrotising gingivitis or periodontitis
Congenital malformations of teeth and enamel	Any malformation of the tooth which is present prior to eruption, including size, shape, texture and position.
Viral, bacterial and fungal infections	Any infection of viral, bacterial or fungal origin that affects the lips and oral cavity
Trauma (including physical and chemical injuries) of the teeth, jawbones and adjacent maxillofacial structures	Physical injury to the teeth, facial bones and adjacent maxillofacial structures
Cysts and tumours of odontogenic origin	Any abnormal mass of tissue or fluid filled cavity that affects the oral cavity or arises from odontogenic tissue origin
Salivary gland disease	Any condition affecting the major or minor salivary glands
Disturbances in the development and growth of oral structures	Any abnormality with respect to timing, aesthetic, physical or functional characteristics of oral structures which is out of the usual limits of normal growth and development.
Mother	A person who has given birth to a child, in this case only biological mothers will be considered to reduce the number of confounding factors
Long term condition	Any condition lasting over 6 months or requiring long term management, this condition may episodic with periods of not being present/affecting the individual between
Pre-natal maternal health condition	Any long term condition that affects a mother prior to the birth of their child
Single condition	Presence/discussion of only one health condition
Comorbidity	Presence of more than one health condition, with a focus on a single, dominant disease
Multimorbidity	Presence of two or more long term health conditions with equal importance given to each
Perinatal complications	Any condition or event that physically affects a mother around the time of childbirth, including in labour. Examples include a traumatic delivery
Post natal health condition	Any long term health condition affecting a mother after the birth of their child
No specified time period	
Tobacco	Consumption of tobacco through any means including cigarette smoking, vaping and smokeless tobacco
Alcohol	Consumption of any alcoholic beverage
Genetic factors	Reference to a genetic characteristic of the mother
Bacterial factors	Reference to any bacterial characteristics of the mother and/or transfer of this to their child
Other	Any other factor not detailed above

The draft extraction tool has been adapted from the JBI extraction tool due to the limited nature of the evidence base demonstrated by the test search, this has led to a broader mapping of the evidence base and an extraction tool to reflect this. It has also been augmented to include information on multimorbidity using key information cited in a systematic review of the measurement of multimorbidity ^20^. The draft data extraction tool will be modified and revised as necessary during the process of extracting data from each included evidence source. Modifications will be detailed in the scoping review. Any disagreements that arise between the reviewers will be resolved through discussion, or with an additional reviewer/s. If appropriate, authors of papers will be contacted to request missing or additional data, where required.

The EPPI-Mapper tool will be used to chart this data into an EGM with the rows and columns shown in Table 3 ( EPPI-Mapper (ioe.ac.uk)). This map will be accompanied by a narrative summary of the evidence guided by the EGM guidance including ^21^;

- Description of the total number of studies,
- Key findings regarding the spread and concentration of evidence across exposure and outcome categories which will allow us to highlight important evidence gaps and trends identified in the research literature,
- Further information regarding study design, population, confidence in study findings assessed using the relevant quality appraisal tool for that study, funding and implementing agency for the included studies,
- The PROGRESS-Plus acronym will be used to assess for the presence of factors associated with health opportunities and outcomes that can lead to inequality such as geographic location, education and ethnicity ^24^,
- Implications and key recommendations for policy and future research,
- A plain language summary highlighting key findings.

## Data Availability

No data are associated with this article.
