# Supplementary material for: The influence of maternal long term health conditions including multimorbidity on child oral health: A scoping review and evidence gap map protocol
Source: Wellcome Open Res. 2024 Nov 8;9:299. Originally published 2024 Jun 10. [Version 2] doi: 10.12688/wellcomeopenres.21725.2 (PMC11568369; doi:10.12688/wellcomeopenres.21725.2)
Supplement: Peer review history

# Reviewer report: Reviewer response for version 2

**DOI:** 10.21956/wellcomeopenres.25787.r110702

**Published:** 2024-11-22

**Authors:** Amit Vasant Mahuli

**Affiliations:** 1 Department of Public Health Dentistry, Rajendra Institute of Medical Sciences, Ranchi, Jharkhand, India

The revised version of the protocol has addressed all the concerns raised. The protocol can be accepted

Is the study design appropriate for the research question?

Partly

Is the rationale for, and objectives of, the study clearly described?

Partly

Are sufficient details of the methods provided to allow replication by others?

Yes

Are the datasets clearly presented in a useable and accessible format?

Partly

Reviewer Expertise:

Oral Health, oral health epidemiology, oral cancer, periodontal health, dental public health

I confirm that I have read this submission and believe that I have an appropriate level of expertise to confirm that it is of an acceptable scientific standard.

# Reviewer report: Reviewer response for version 2

**DOI:** 10.21956/wellcomeopenres.25787.r110889

**Published:** 2024-11-15

**Authors:** Anwar Merchant

**Affiliations:** 1 University of South Carolina, Columbia, USA

The authors propose to conduct a scoping review to evaluate the evidence linking maternal long term health conditions and child oral health.

 The aims could be made more explicit in the introduction. Specifically, what do the newer theories posit? Which are the most important factors shared by the mother that drive child oral health? These could include untreated caries in the mother, household food insecurity, access to dental care, water fluoridation, and preventive oral health services in primary care. These factors can interact with management of chronic disease in the mother with oral health consequences in the child.

Is the study design appropriate for the research question?

Yes

Is the rationale for, and objectives of, the study clearly described?

Partly

Are sufficient details of the methods provided to allow replication by others?

Yes

Are the datasets clearly presented in a useable and accessible format?

Yes

Reviewer Expertise:

Evaluating the connections between diet, oral conditions and treatment and systemic health.

I confirm that I have read this submission and believe that I have an appropriate level of expertise to confirm that it is of an acceptable scientific standard.

# Reviewer report: Reviewer response for version 1

**DOI:** 10.21956/wellcomeopenres.23999.r98124

**Published:** 2024-10-09

**Authors:** Ratna Indriyanti

**Affiliations:** 1 Department of Pediatric Dentistry, Faculty of Dentistry, Universitas Padjadjaran, Indonesia, Indonesia

As a type of literature review, the method  preparation meets the requirements as a scoping review, where the method used can get a broad picture of the topic you want to explore widely without strict limitations.

 Likewise, the topic can be mapped comprehensively.

 However, there are several things that need attention:

 1. The title listed does not adequately describe the splendor and depth of the research carried out.

 2. Provide a more detail explanation regarding health condition in the oral cavity other than caries.

 3, The alleged relationship between multimorbidity and childrens oral health has not been clearly described.

Is the study design appropriate for the research question?

Yes

Is the rationale for, and objectives of, the study clearly described?

Yes

Are sufficient details of the methods provided to allow replication by others?

Yes

Are the datasets clearly presented in a useable and accessible format?

Yes

Reviewer Expertise:

kraniofacial growth and development

I confirm that I have read this submission and believe that I have an appropriate level of expertise to confirm that it is of an acceptable scientific standard.

# Response

**Published:** 2024-11-06

**Authors:** Faith Campbell

**Affiliations:** School of Dentistry, University of Dundee, Dundee, Scotland, UK

1. The title listed does not adequately describe the splendor and depth of the research carried out.

**Response: **Thank you, the title has been updated to ‘The Influence of Maternal Health Factors Long Term Health Conditions Including Multimorbidity on Child Oral Health: A Scoping Review and Evidence Gap Map Protocol’, to better reflect the scope of this review (page 1)

 2. Provide a more detail explanation regarding health condition in the oral cavity other than caries.

**Response: **Thank you, we agree that oral health conditions could have been better detailed and hope that the addition of the following paragraph satisfies this (page 3 paragraph 1) ‘Dental caries will be the primary outcome focus of this scoping review. This is because the work which this review will inform will focus on data on child dental caries in Scotland. Consideration will also be given to conditions of enamel that increase tooth susceptibility to enamel such as molar incisor hypomineralisation (MIH) and developmental defects of enamel (DDE) as they may be causative pathways in any link between maternal health conditions and childhood caries. Thereafter, in this review ‘oral health will be defined as dental caries, MIH and DDE. Other oral conditions in children will not be considered due to an absence of theoretical links between them and maternal health conditions. ‘

 3, The alleged relationship between multimorbidity and childrens oral health has not been clearly described.

**Response: **Due to this being a novel review, there is no evidence that describes a relationship between multimorbidity and child oral health. This gap in knowledge forms the need for the review. We have described current theories on possible links in the following paragraphs   (Page 3 paragraphs 2 and 3) ‘ **Possible link’** There has been a demonstrated link between poor oral health, socio-economic inequalities and co-morbidities including diabetes, cardiovascular disease, rheumatoid arthritis, chronic obstructive pulmonary disease (COPD), pneumonia in the elderly, anxiety and depressive disorders, and increased risk of developing cognitive impairment and dementia (3). This link is frequently bi-directional. However, the link between multimorbidity and oral health, particularly dental caries, is less researched (3).  Multimorbidity and poor oral health share some common risk factors such as deprivation: both dental caries and multimorbidity disproportionately affect those living in more deprived areas (4, 5). The increasing number of people living with multimorbidity, results in an increasing need for new models of dental care to address multimorbidity in relation to adult oral health (3). Individuals with multimorbidity have a greater treatment burden than the general population, whereby they need to access treatment from multiple (often uncoordinated) healthcare professionals (3). These care plans are often complex and those with multimorbidity are often taking multiple medications (3). Best care for these patients requires appropriate training of oral health professionals, changes to the practice and delivery of oral health care and a focus of research on multimorbidity and oral health (3). **(Page 3 paragraph 4)** **Current Evidence** Initial explorations indicate that there is a limited but evolving body of evidence regarding child oral health and child or maternal long term health conditions. Newer theoretical models are emerging that detail the many factors that can influence child oral health at child, family and community levels (6). The association between maternal long term health conditions and child oral health has been explored, with preliminary evidence suggesting a link between shared environmental factors and direct maternal-to-child transfer of bacteria, diet and substance use during pregnancy causing childhood caries (7, 8). Maternal long term health conditions may additionally create challenges in supporting child oral care and in attending dental appointments (6). To date, no review has synthesised this evidence to examine the relationship between maternal long term health conditions and child oral health.

# Reviewer report: Reviewer response for version 1

**DOI:** 10.21956/wellcomeopenres.23999.r98125

**Published:** 2024-09-23

**Authors:** Amit Vasant Mahuli

**Affiliations:** 1 Department of Public Health Dentistry, Rajendra Institute of Medical Sciences, Ranchi, Jharkhand, India

- The study protocol, influence of maternal health factors, including multimorbidity on child oral health, is a promising topic to explore the maternal factors and their indirect and direct influences on the oral health of the child.
- The rationale of the study can be more clearly stated as the maternal multi-morbidity is vague as per the definition, and the use of term child oral health is also a high age range that can lead to heterogenicity of the data extracted. As different age groups will have different oral health needs and oral health issues.   
- The studies included in the scoping review and their quality assessment, if done, will be challenging as many study designs will be included.
- The data showing severe periodontal disease could be challenging as their are more reported cases of gingival disease than severe periodontal disease in children. 
- The protocol fits in to the scoping review category, but the rationale, condition, and assessment are going to be challenging as the data obtained will be difficult to fit in the data charting tool.

Is the study design appropriate for the research question?

Partly

Is the rationale for, and objectives of, the study clearly described?

Partly

Are sufficient details of the methods provided to allow replication by others?

Yes

Are the datasets clearly presented in a useable and accessible format?

Partly

Reviewer Expertise:

Oral Health, oral health epidemiology, oral cancer, periodontal health, dental public health

I confirm that I have read this submission and believe that I have an appropriate level of expertise to confirm that it is of an acceptable scientific standard, however I have significant reservations, as outlined above.

# Response

**Published:** 2024-11-06

**Authors:** Faith Campbell

**Affiliations:** School of Dentistry, University of Dundee, Dundee, Scotland, UK

The study protocol, influence of maternal health factors, including multimorbidity on child oral health, is a promising topic to explore the maternal factors and their indirect and direct influences on the oral health of the child.

**Response: **Thank you for your feedback, we are pleased that you agree on the importance of this topic.

 The rationale of the study can be more clearly stated as the maternal multi-morbidity is vague as per the definition, and the use of term child oral health is also a high age range that can lead to heterogenicity of the data extracted. As different age groups will have different oral health needs and oral health issues. 

**Response: **There are challenges when studying multimorbidity due to heterogenous definitions across literature which is why ‘health conditions’ were selection for this review.   Thank you, we have clarified that in this review child will mean an individual below the age of 18 per the United Nations and Scottish Government definitions on page 2 paragraph 6.   If evidence can be explored by age group this will be discussed within the paper (page 2 paragraph 6)

 The studies included in the scoping review and their quality assessment, if done, will be challenging as many study designs will be included.

**Response: **We agree that such a heterogenous evidence base may be more challenging to appraise however since the pilot search revealed such limited studies for inclusion, it was decided to include all available evidence to provide as much evidence as possible.

 The data showing severe periodontal disease could be challenging as their are more reported cases of gingival disease than severe periodontal disease in children. 

**Response: **We also agree that being such a rare outcome, severe periodontal disease will be scarce. Periodontal disease and gingival conditions in children will not be included as an outcome in this review.

 The protocol fits in to the scoping review category, but the rationale, condition, and assessment are going to be challenging as the data obtained will be difficult to fit in the data charting tool.

**Response: **We agree that the data obtained will require amendments to the traditional data charting tools used for scoping review and considered this when developing our protocol. We have therefore amended the charting tool to reflect this and will iteratively develop this to best extract data from the studies that are included, per JBI protocol for scoping reviews (page 11).

# Reviewer report: Reviewer response for version 1

**DOI:** 10.21956/wellcomeopenres.23999.r86723

**Published:** 2024-07-15

**Authors:** Betsy Foxman

**Affiliations:** 1 University of Michigan,, Ann Arbor, USA

The authors propose conducting a scoping review to explore the current evidence between maternal multimorbidity and child oral health. They outline standard procedures for conducting such a review.

 My 'partly' responses reflect :

 1) There is no definition or inclusion criteria regarding child oral health (this is a fatal flaw).

 2) Lack of details on the types of maternal oral health and other morbidities that will be included as multimorbidities. It is likely there will be sufficient literature on gum disease and dental caries but little on injury or factors such as tongue rings. Similarly, the literature on oral health and diabetes and heart disease is robust but limited on factors such as sexually transmitted bacterial or viral infections in the oral cavity.  

 The background mentions the importance of shared environmental factors and social inequalities, but these factors are not identified in the research questions nor in the data collected.  Taking those factors into account will be particularly important for cross-cultural comparisons.

Is the study design appropriate for the research question?

Partly

Is the rationale for, and objectives of, the study clearly described?

Yes

Are sufficient details of the methods provided to allow replication by others?

Partly

Are the datasets clearly presented in a useable and accessible format?

Yes

Reviewer Expertise:

Molecular epidemiology of dental caries;  molecular epidemiology of bacterial infections and the oral microbiome

I confirm that I have read this submission and believe that I have an appropriate level of expertise to state that I do not consider it to be of an acceptable scientific standard, for reasons outlined above.

# Response

**Published:** 2024-11-06

**Authors:** Faith Campbell

**Affiliations:** School of Dentistry, University of Dundee, Dundee, Scotland, UK

1) There is no definition or inclusion criteria regarding child oral health (this is a fatal flaw).

**Response:  **Thank you for highlighting this. This has been addressed by adding the following paragraph to the background (page 2 paragraph 7) ‘ *Dental caries will be the primary outcome focus of this scoping review. This is because the work which this review will inform will focus on data on child dental caries in Scotland. Consideration will also be given to conditions of enamel that increase tooth susceptibility to enamel such as molar incisor hypomineralisation (MIH) and developmental defects of enamel (DDE) as they may be causative pathways in any link between maternal health conditions and childhood caries. Thereafter, in this review ‘oral health will be defined as dental caries, MIH and DDE. Other oral conditions in children will not be considered due to an absence of theoretical links between them and maternal health conditions.’ * The inclusion and exclusion criteria have been refined to reflect this (page 5 paragraph 4) ‘ *Condition* *Inclusion criteria: *

- *Exposure: single or multiple health conditions (multimorbidity) affecting mothers at any time from prior to conception to the child being 16 years old.*

*Outcome: Child oral health defined as dental caries, MIH and DDE Exclusion: *

- *Exposure: No clear definition of health condition that is affecting mother  *

*Outcome:  Dental trauma, malocclusion and gingivitis’*

 2) Lack of details on the types of maternal oral health and other morbidities that will be included as multimorbidities. It is likely there will be sufficient literature on gum disease and dental caries but little on injury or factors such as tongue rings. Similarly, the literature on oral health and diabetes and heart disease is robust but limited on factors such as sexually transmitted bacterial or viral infections in the oral cavity.  

**Response: **Thank you for your comment. For clarity this review will not be exploring multimorbidity due to the paucity in evidence revealed by the pilot search for this review. We hope that this is clarified by the rephrasing of the following paragraph (page 4 paragraph 3) ‘The objective of this scoping review is to map existing literature on the relationship between maternal long-term health conditions, and child oral health. Maternal long term health conditions have been chosen because an initial pilot search found such limited evidence regarding maternal multimorbidity as a concept, that a broader search to map the evidence base on maternal long term health conditions and child oral health would provide greater value. This will subsequently inform research regarding the presence of multiple long term health conditions (multimorbidity) and child oral health.’ We have decided to exclude behaviour and lifestyle choices as this is much more challenging to explore, so within a field which is sparsely researched thus far, we felt it more important to explore health conditions. Furthermore these health conditions are examinable in the datasets which we will use in later stages of this project, unlike factors such as nose ring wearing. We agree that bacterial transmission from mother to child should be considered. As this wider programme of work focusses on multiple long term conditions (multimorbidity), only long terms conditions will be explored. We are aware of the significant body of research on bacteria in mothers’ oral cavity and the impact of transmission of this to children with a theory of caries transmission, such as strep mutans. Given that it is widely accepted within the scientific community that caries is caused by dysbiosis in the oral cavity rather than transmission of a specific commensal, we made the decision to exclude bacterial transmission from mother to child in this review. Multiple papers discussing this were found in the initial search and therefore this will be discussed in greater detail in the discussion of results.  

 3) The background mentions the importance of shared environmental factors and social inequalities, but these factors are not identified in the research questions nor in the data collected.  Taking those factors into account will be particularly important for cross-cultural comparisons.

**Response: **We agree that shared environmental factors and social inequalities are both important when considering dental caries in children and multiple long-term conditions in mothers. We have not included them in this review as the evidence is so indisputable on this that we felt it was most important to focus on what is not known with such certainty regarding causes of caries in children (1, 2). However, deprivation and social inequalities will be key confounders in our future research exploring mothers’ health data and childhood caries.
